# Bridging endoscopic pancreatic stenting for disconnected pancreatic duct syndrome using a rendezvous technique from a walled-off necrosis cavity

**DOI:** 10.1055/a-2219-2672

**Published:** 2024-01-09

**Authors:** Shuntaro Mukai, Takao Itoi, Atsushi Sofuni, Takasyoshi Tsuchiya, Reina Tanaka, Ryosuke Tonozuka, Hiroyuki Kojima

**Affiliations:** 1 Department of Gastroenterology and Hepatology, Tokyo Medical University, Tokyo, Japan

Disconnected pancreatic duct syndrome (DPDS) is sometimes complicated by walled-off necrosis (WON) and is associated with a prolonged disease course 1 2 . Treatment with endoscopic pancreatic stenting to bridge the disconnected pancreatic duct is useful, but it is technically challenging in many cases 3 4 5 . Here, we report a case of successful bridging with an endoscopic pancreatic stent for DPDS using the rendezvous technique from a WON cavity into which a lumen-apposing metal stent (LAMS) was placed.

A 46-year-old woman had infected WON complicated by necrotizing pancreatitis due to hyperparathyroidism. The WON was multilocular and forming cavities in the pancreatic head and body ( Fig. 1 ). Furthermore, the pancreatic duct was disrupted in the pancreatic body, and disconnected in the pancreatic head ( Fig. 2 **a** ).

**Fig. 1:**
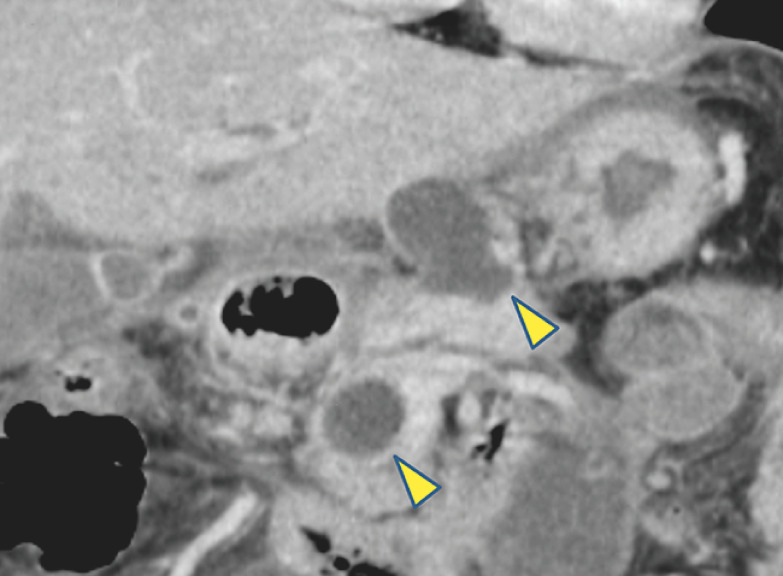
Radiographic image showing the multilocular walled-off necrosis forming cavities in the pancreatic head and body (arrows).

**Fig. 2:**
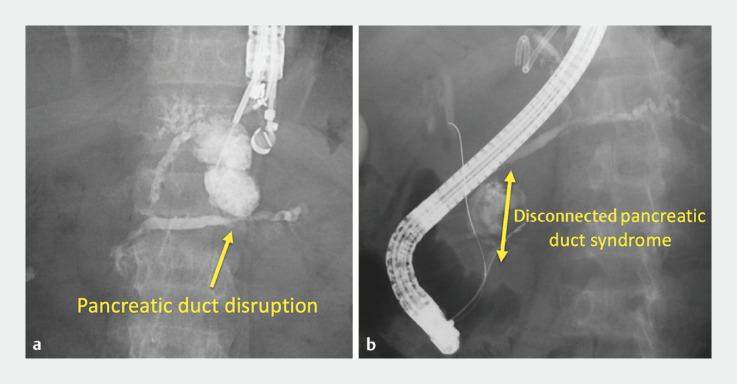
Fluoroscopic images showing: **a** the disruption of the pancreatic duct in the pancreatic body, as seen during endoscopic ultrasound-guided transmural drainage; **b** disconnected pancreatic duct syndrome in the pancreatic head, with access to the tail side of the pancreatic duct proving impossible.

First, endoscopic ultrasound (EUS)-guided transmural drainage was performed using an electrocautery-enhanced LAMS system to drain the infected pancreatic body cavity. The infection improved, but the patient’s symptoms of abdominal pain associated with pancreatic leakage did not improve. Transpapillary bridging endoscopic pancreatic stenting was therefore attempted to repair the pancreatic duct disruption; however, the approach to the tail side of the pancreatic duct across the disconnected pancreatic duct at the pancreatic head was unsuccessful ( Fig. 2 **b** ).

A rendezvous technique was then attempted by approaching from the pancreatic duct disruption ( Video 1 ). An upper gastrointestinal endoscope was inserted into the previously placed LAMS to identify the point of pancreatic duct disruption. From here, it was possible to insert a guidewire across the disconnected pancreatic duct into the duodenum through the minor papilla. A duodenoscope was inserted, and biopsy forceps were used to grasp the guidewire and pull it into the scope ( Fig. 3 **a** ). A double-lumen catheter was inserted alongside the guidewire, and a second guidewire was inserted into the tail side of the pancreatic duct. After balloon dilation of the minor papilla had been performed, a bridging pancreatic duct stent was successfully placed ( Fig. 3 **b** ). The patient’s symptoms of abdominal pain improved after this successful endoscopic treatment.

**Fig. 3:**
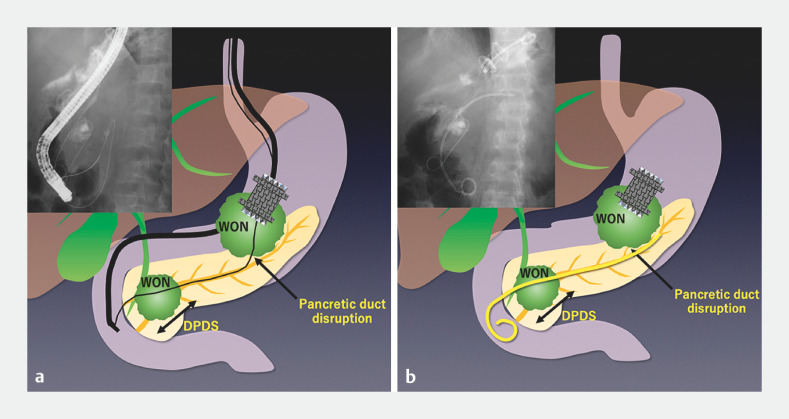
Schemas of bridging endoscopic pancreatic stenting for disconnected pancreatic duct syndrome using a rendezvous technique from the walled-off necrosis (WON) cavity showing: **a** a guidewire inserted from the point of duct disruption into the duodenum via the minor papilla; **b** a bridging pancreatic duct stent placed across the disconnected pancreatic duct in the pancreatic head and the disrupted part of the pancreatic body.

A bridging endoscopic pancreatic stent is inserted for disconnected pancreatic duct syndrome using a rendezvous technique from a walled-off necrosis cavity via the lumen-apposing metal stent that had been previously placed during endoscopic ultrasound-guided transmural drainage. Source for stent: Boston Scientific.Video 1

Endoscopy_UCTN_Code_TTT_1AS_2AD

## Notes

Endoscopy E-Videos https://eref.thieme.de/e-videos
*E-Videos* is an open access online section of the journal *Endoscopy* , reporting on interesting cases and new techniques in gastroenterological endoscopy. All papers include a high-quality video and are published with a Creative Commons CC-BY license. Endoscopy E-Videos qualify for HINARI discounts and waivers and eligibility is automatically checked during the submission process. We grant 100% waivers to articles whose corresponding authors are based in Group A countries and 50% waivers to those who are based in Group B countries as classified by Research4Life (see: https://www.research4life.org/access/eligibility/ ). This section has its own submission website at https://mc.manuscriptcentral.com/e-videos .
